# IL-10 Producing Regulatory B Cells Mediated Protection against Murine Malaria Pathogenesis

**DOI:** 10.3390/biology11050669

**Published:** 2022-04-27

**Authors:** Meenu Kalkal, Rubika Chauhan, Reva Sharan Thakur, Mrinalini Tiwari, Veena Pande, Jyoti Das

**Affiliations:** 1 Parasite-Host Biology, ICMR-National Institute of Malaria Research, Dwarka, New Delhi 110077, India; meenukalkal@gmail.com (M.K.); rubika50chauhan@gmail.com (R.C.); thakurreva@gmail.com (R.S.T.); mrinalini.tiwari22@gmail.com (M.T.); 2 Biotechnology Department, Kumaun University, Nainital 263001, India; veena.biotech@gmail.com

**Keywords:** *Plasmodium*, B-cells, regulatory B cells (Bregs), malaria, interleukin-10 (IL-10), immunomodulation

## Abstract

**Simple Summary:**

The immunomodulatory role of B cell subset called regulatory B cells was evaluated during *Plasmodium* infection to study their role in susceptibility or resistance during infection. The expansion of regulatory B cells during *Plasmodium* infection indicated their important role in regulating the immune response. Adoptive transfer of regulatory B cells following infection with a lethal parasite resulted in enhanced survival of mice and inhibited growth of the *Plasmodium* parasite. Moreover, by inhibiting the production of the pro-inflammatory cytokine, IFN-γ, and stimulating anti-inflammatory IL-10 production, regulatory B cells may serve as an important contributor to protective immune response.

**Abstract:**

Various immune cells are known to participate in combating infection. Regulatory B cells represent a subset of B cells that take part in immunomodulation and control inflammation. The immunoregulatory function of regulatory B cells has been shown in various murine models of several disorders. In this study, a comparable IL-10 competent B-10 cell subset (regulatory B cells) was characterized during lethal and non-lethal infection with malaria parasites using the mouse model. We observed that infection of Balb/c mice with *P. yoelii* I 7XL was lethal, and a rapid increase in dynamics of IL-10 producing B220^+^CD5^+^CD1d^+^ regulatory B cells over the course of infection was observed. However, animals infected with a less virulent strain of the parasite *P. yoelii* I7XNL attained complete resistance. It was observed that there is an increase in the population of regulatory B cells with an increase of parasitemia; however, a sudden drop in the frequency of these cells was observed with parasite clearance. Adoptive transfer of regulatory B cells to naïve mice followed by infection results in slow parasite growth and enhancement of survival in *P. yoelii* 17XL (lethal) infected animals. Adoptively transferred regulatory B cells also resulted in decreased production of pro-inflammatory cytokine (IFN-γ) and enhanced production of anti-inflammatory cytokine (IL-10). It infers that these regulatory B cells may contribute in immune protection by preventing the inflammation associated with disease and inhibiting the parasite growth.

## 1. Introduction

Malaria represents the most significant public health problem across the globe. In tropical countries, it is one of the principal causes of morbidity and mortality. The world malaria report 2021 estimated that 241 million malaria cases occurred during 2020 [1]. The malaria parasite is known to have a very complex multistage life cycle that is responsible for the generation of a robust immune response. Pro-inflammatory and anti-inflammatory immune responses are triggered at the blood stage of parasite infection [2]. A pro-inflammatory response is characterized by increased secretion of IFN-γ that prevents the growth of the parasite [3]. However, advanced or excessive pro-inflammatory response sometimes cause tissue pathology in the host. On the other hand, the anti-inflammatory response is mainly characterized by the release of IL-10, an anti-inflammatory cytokine, known to have a counter-effect on the inflammatory response by preventing the tissue pathology [4,5]. Therefore, a subtle balance of pro- and anti-inflammatory responses is crucial for establishing effective immune responses during infection [6,7]. In addition, the critical balance of the immune signaling pathway is also regulated by immune checkpoint activation. A notable mediator of the immune checkpoint is the association of PD-1 with its ligand, PD-L1. Binding of PD-1/PD-L1 regulates the activation of T and B-cells, subsequently suppressing the T and B-cell mediated immune response. Although, the interaction of PD-1/PD-L1 is indispensable for self-tolerance and autoimmunity, its early activation may hinder the immune response by inhibiting the activation of T and B-cells [8]. Reciprocally, a number of studies describe that abrogation of such PD-1/PD-L1 interactions reinstate the function of T and B cells and ultimately contribute to protection [9,10]. Nonetheless, several pieces of evidence indicate that the host immune response against malaria parasite modulates throughout infection, and different immune components of the adaptive immune system are involved in providing protection [11,12,13]. Besides, different species of *Plasmodium* parasites are also known to activate the host’s immune system in different ways.

Interleukin-10 (IL-10), is an important anti-inflammatory cytokine well known to limit the excessive inflammatory immune response induced due to infection by pathogen and thereby have protective effect on host [14,15]. Earlier production of IL-10 was confirmed in T-helper 2 cells. Later, the production of IL-10 was evidenced by several other cells of innate as well as an adaptive immune system like Th1 cells, Treg cells, Th17 cells, Tfh cells, CD8^+^ T cells, B cells, and regulatory B cells. Besides B and T cells, dendritic cells (DCs), natural killer (NK) cells, macrophages, mast cells, eosinophils, and neutrophils are also known to produce IL-10 [16,17]. Depending on the immune response and strain of the *Plasmodium* parasite, IL-10 can be considered a friend or foe. Several recent studies using murine experimental models have identified the importance of IL-10 during *Plasmodium* infection [5,18,19]. In addition, multiple pieces of evidence for the increased IL-10 level in the serum of malaria patients are available [2,20,21]. Recently, the immunoregulatory function of splenic B cells via the production of IL-10 has been demonstrated in a variety of autoimmune diseases, cancer, organ transplantation, and various infectious diseases [22,23]. The development and activation of regulatory B cells depend on the infection microenvironment in both humans and mice [24]. However, the importance of IL-10^+^ B-cells in the regulation of immune response and immunopathology in malaria has not been explored so far to an extent [25,26]. With limited evidence, regulatory B cells (Bregs) have been demonstrated to perform an immunomodulatory role during *Plasmodium* infection [25,26], and it seems to be dependent on parasite species and their pathogenicity. However, substantial development has not been made in the characterization of regulatory B cells in malaria pathogenesis. Further, the role of these regulatory B cells during lethal and non-lethal malaria, their cell surface markers that are unique to these regulatory B cells, also have not been specified in both murine as well as human malaria. Therefore, this novel subset of B cells needs to be explored during malaria pathogenesis to attain more insights into their role in susceptibility or resistance during infection. The present study investigated the dynamics and expansion of regulatory B cells and their role during lethal and non-lethal *Plasmodium yoelii* parasite infection. In addition, we also studied the impact of these regulatory B cells by adoptive transfer in *Plasmodium yoelii* 17XL infected mice. Results from our study demonstrate that regulatory B cells are a vital novel subset of B cells required to protect against the parasite by modulating the immune response.

## 2. Materials and Methods

Animals: In this study, Balb/c (Female) mice, aged 6- to 8-weeks, were reared in the institute animal facility under a hygienic and specific pathogen-free environmental conditions. All animals used were approved for experiments (Approval number: IAEC/NIMR/2020-1/02) by Institutional Animal Ethics Committee (IAEC) in accordance with the guidelines of the Committee for the Purpose of Control and Supervision of Experiments on Animals (CPCSEA).

Parasite infection in mice: Cryopreserved *Plasmodium yoelii* 17XL and *Plasmodium yoelii* 17XNL were received BEI resources (USA). Both lethal and non-lethal strains of the *Plasmodium* parasite were passaged from one mouse to another before use in experiments. For experimental purposes, mice were infected with 5 × 10^5^ syngeneic parasitized red blood cells (pRBCs) by intra-peritoneal injection.

Parasitemia determination: The progression of infection at different time points was determined by calculating the percentage of parasitaemia in *P. yoelii* 17XL and *P. yoelii* 17XNL infected animals. For this, thin blood films were prepared on glass slide by bleeding via tail vein of experimental mice. Blood smears were fixed in methanol followed by staining with Giemsa stain (Sigma–Aldrich, St. Louis, MO, USA). Parasitemia was calculated by counting the percentage of infected cells per 5000 red blood cells (RBCs) in each glass slide. The parasitemia is generally defined as the mean percentage of pRBC± standard deviation (SD) of the mean in each group of the mice.

Cell preparation: spleen of normal and infected Balb/c mice were harvested post malaria infection at different times. The single-cell suspension of splenocytes was made in phosphate buffered saline (Thermo Fisher Scientific, Waltham, MA, USA) by squashing the spleens between frosted slides isolated from control and infected mice. Spleen cells were further collected by centrifuging the suspension for 10 min at 2000 rpm. Red Blood Cells (RBCs) were lysed with RBC lysis buffer (0.15 M NH_4_Cl, 10 mM KHCO_3_, 0.1 mM Na_2_EDTA) and the cells were washed three times in a fresh medium. Cell count was determined and cell viability was measured by Trypan Blue Dye exclusion (Thermo Fisher Scientific, Waltham, MA, USA). After washing, single cells suspension was prepared using complete RPMI 1640 (Thermo Fisher Scientific, Waltham, MA, USA) medium.

Flow cytometric staining and analysis: Splenocytes (10^7^ cells/mL) were prepared using FACS buffer (PBS, 3% FCS, 0.01% Na-azide) for cell surface staining. To characterize the surface phenotype of the cell lines, cells were incubated with different combinations of following specific fluorescent antibodies for CD4 (Percp, Clone: RM4-5), CD8 (AF700, 53-6.7), B220/CD45R (APC, Clone: RA3-6B2), CD5 (FITC, Clone: 53-7.3), CD1d (BV421, Clone: 1B1), PD-L1 (PE, Clone: MIH5) for 30 min at 4 °C in the dark as described in a previous study [27]. Briefly, cells were washed twice with staining buffer at 1500 rpm for 5 min and fixed with 1% paraformaldehyde. All antibodies used in the experiments were procured from BD Biosciences (Franklin Lakes, NJ, USA). An LSR Fortessa (BD Biosciences, Franklin Lakes, NJ, USA) flow cytometer was used for acquisition of cells and data was analyzed by using FlowJo software v10 (Tree Star, Ashland, OR, USA).

For intracellular staining, splenocytes were re-suspended at a concentration of 2 × 10^6^ cells/mL in complete medium and incubated overnight in 12 well plate for activation with 1 µg/mL anti-CD3 (Clone: 145-2C11) and 2 µg/mL of anti-CD28 (Clone: 37.51). After overnight incubation, GolgiPlug protein transport inhibitor (BD Biosciences, Franklin Lakes, NJ, USA) was added (1 µg/mL) during the last 2 h of incubation to inhibit cytokine secretion as described previously [28]. For cell-surface staining, splenocytes at a concentration of 10^7^ cells/mL were suspended in staining buffer (PBS, 3% FCS), and 30 µL of cell suspension was incubated with fluorescent conjugated antibodies for a duration of 30 min at 4 °C under dark conditions. Next, stained cells were washed twice with staining buffer at 1500 rpm for 5 min. Surface-stained cells were fixed with BD Cytofix/Cytoperm according the manufacturer’s instructions. Further, the cells were washed twice with PBS and washed cells were suspended again in a permeabilization buffer (Cytofix/Cytoperm kit; BD Biosciences Franklin Lakes, NJ, USA) for permeablisation. For intracellular staining, these cells were stained with fluorescently conjugated staining antibodies for IL-10 (PE, Clone: JES516E3), IFN-γ (PE, Clone: XMG1.2). Fluorescent intensity was acquired with an LSR Fortessa.

Histological examination and hemozoin quantification: Spleens obtained from *P. yoelii* infected and wild-type control mice were processed for histopathological examination and quantification of hemozoin content at different days post-infection. For histological study, a small part of each spleen was fixed in 4% paraformaldehyde and subsequently these formalin fixed tissues were embedded in paraffin followed by cutting into 5-mm thin sections. These sections were examined using bright-field microscopy coupled with hematoxylin and eosin (H&E) staining according to standard protocols. The results were confirmed by observation of at least than ten fields per group in a double-blinded manner. The other half part of spleen was cryogenically stored until further analysis. Further cryogenically stored spleen tissues were thawed and processed for quantification of hemozoin content as described by Pisciotta et al. [29]. In brief, spleen isolated from infected and control mice were homogenized and lysed in 5 mL of deionized H_2_O using sonicator. To pellet hemozoin, lysed spleen tissue samples were centrifuged at 14,000× *g* for 15 min and supernatant was removed. Hemozoin pellets were resuspended in 1 mL solution containing 2% SDS, 100 mM sodium bicarbonate and again centrifuged at 14,000× *g* for 15 min. Pellets were washed twice with 2% SDS and washed pellets were resuspended again and incubated overnight in 1 mg/mL proteinase K Buffer at 60 °C. After incubation, pellets were washed with deionized H_2_O and the purified hemozoin pellets were solubilized in 1ml solution of 2% SDS and 20 mM NaOH for 1 h. Finally, all samples were quantified spectrophotometrically at absorbance of 400 nm with molar extinction coefficient of 1 × 10^5^. All reagents used for hemozoin quantification were procured from Sigma–Aldrich, St. Louis, MO, USA.

Isolation and stimulation of regulatory B cells: IL-10-producing B cells were isolated according to the manufacturer’s instructions using a regulatory B cell isolation kit (Miltenyi Biotec, Bergisch Gladbach, Germany). First, single-cell suspension of splenocytes was prepared from spleen of *Plasmodium yoelii* 17XL infected mice. B cells were enriched from total splenocytes by negative selection using biotin labelled antibodies cocktail provided with the kit. The pre-enriched B cells were cultured at 2 × 10^6^ cells/mL in complete medium and stimulated for 5 h with PMA (10 ng/mL) and ionomycin (500 ng/mL) purchased from Sigma–Aldrich, St. Louis, MO, USA. Next, cells were harvested and further incubated with IL-10 catching reagent to allow binding to IL-10 secreting cells at 37 °C for 45 min. Finally, the IL-10^+^ and IL-10^−^ B cells were enriched with anti-IL-10 magnetic microbeads. The purity of the isolated IL-10^+^ and IL-10^−^ B cell populations was determined by using flow cytometry.

Adaptive transfer of regulatory B cells: 5 × 10^5^ regulatory B cells were diluted in 200 µL PBS and transferred into recipient naive mice intravenously (i.v.) followed by infection with *Plasmodium yoelii* 17XL. As a control, IL-10^−^ B cells were adoptively transferred to a different group of mice.

Serum analysis: Serum samples were collected and analyzed for level of cytokines in serum by multiplexed bead array immunoassay using Luminex technology (Bio-Plex; Bio-Rad laboratories, Hercules, CA, USA) according to manufacturers’ protocol.

Statistical analysis: All statistical analyses were performed in Microsoft Excel 2019 MSO (Version 2202 Build 16.0.14931.20116). A student’s *t*-test was used to analyze the significance of differences between the two groups, and *p* value ≤ 0.05 were considered significant. Quantitative data are depicted as the mean ± SD.

## 3. Results

### 3.1. Parasitaemia and Survival during Lethal and Non-Lethal Plasmodium yoelii Infection

**Figure 1:**
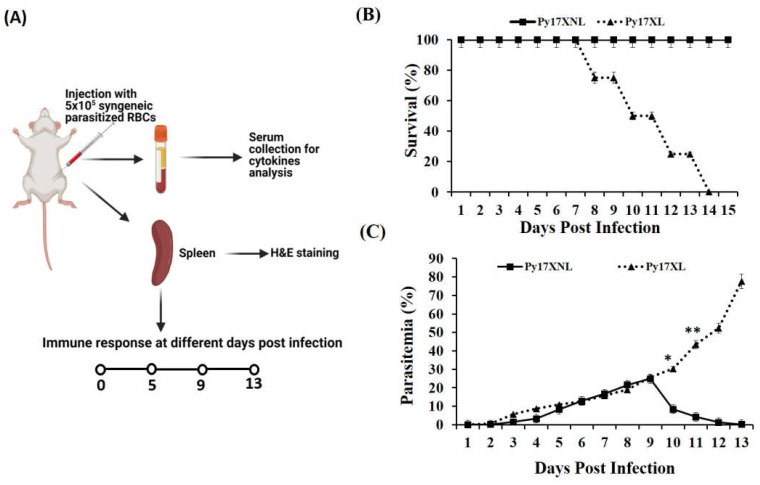
Parasitaemia and survival during lethal and non-lethal *Plasmodium yoelii* infection: (**A**) Balb/c mice were infected with 5 × 10^5^ *Plasmodium yoelii* 17XL and *Plasmodium yoelii* 17XNL parasitized RBCs via intra- peritoneal (i.p) injection. Malaria parasites infected mice were monitored for immune response, survival and parasitemia. (**B**) The survival rate of mice infected with *Py* 17XL (triangle, *n* = 12) and *Py* 17XNL (square, *n* = 12). Data is shown as mean ± SD from 12 mice pooled from three independent experiments. (**C**) Thin blood smears were prepared from the tail vein at various time points as indicated in the figure and stained with Giemsa stains. Parasitemia was determined by counting the percentage of infected cells per 5000 RBCs per slide. Parasitemia of lethal *Py* 17XL (triangle, *n* = 12) and non-lethal *Py* 17XNL (square, *n* = 12) at different time points. The unpaired student’s *t*-test was used to determine the statistical significance (** *p* < 0.001, * *p* < 0.01).

Female Balb/c mice were infected with *Plasmodium yoelii* lethal (*Py* 17XL) and non-lethal (*Py* 17XNL) strains. *Py* 17XL and *Py* 17XNL are known to possess different virulency that results in different types of immune response (Figure 1A). After infection, parasitized erythrocytes appeared in blood at 3rd day post-infection (p.i.) and their number keeps on increasing continuously until day 9 post-infection for both strains. *Plasmodium yoelii* 17 XNL has a slow growth rate, and the infection from blood gets cleared after achieving the peak parasitaemia within 15 days post-infection. Therefore, 100% of infected animals survived until the experiment termination implying the non-lethal nature of the *Py* 17XNL *Plasmodium* parasite (Figure 1B). However, *Plasmodium yoelii* 17XL is a lethal strain that multiplies rapidly, and infected animals generally die within two weeks after injection of the parasite. Moreover, a continuous increase in parasitaemia in animals infected with the *Py* 17XL strain of malaria parasite was observed and eventually they died on 13–15th days post-infection (Figure 1C).

### 3.2. Spleen Histopathology and Hemozoin Content Analysis during Py 17XL and Py 17XNL Infection

**Figure 2:**
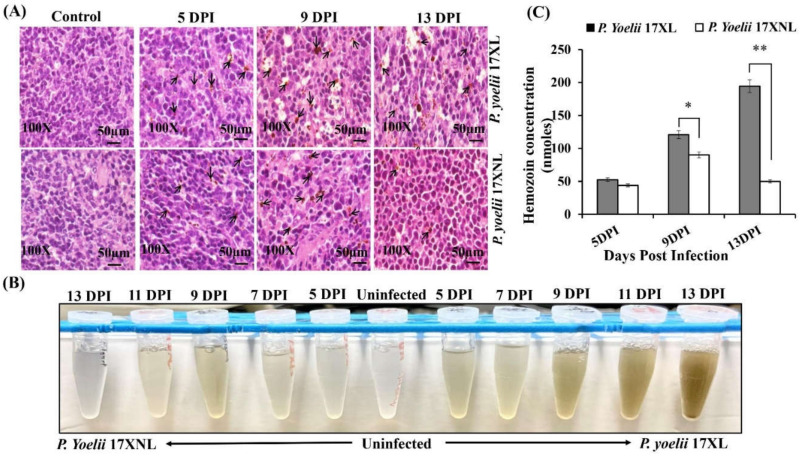
Spleen histopathology and hemozoin quantification during *Py* 17XL and *Py* 17XNL infection: Balb/c mice were infected with *Py* 17XL and *Py* 17XNL parasites. At indicated time points, mice were sacrificed and spleen tissue was processed for histological examination and hemozoin quantification. (**A**) The presence of hemozoin crystals was monitored in the spleen sections of both *P. yoelii* 17XL and *P. yoelii* 17XNL infection at different time points. Black arrows specify the hemozoin content at original magnification of 100X in the spleen of Balb/c mice infected with *P. yoelii* 17XL and *P. yoelii* 17XNL at 5th, 9th, and 13th day post-infection. (**B**) Extracted hemozoin from 60–70 mg of spleen tissues obtained after infection at different indicated times. (**C**) Extracted hemozoin was quantified spectrophotometrically in spleen of mice infected with *Py* 17XL and *Py* 17XNL and expressed as nmoles/spleen. Unpaired two-tailed Student’s *t*-test was used to calculate the statistical significance (** *p* < 0.001, * *p* < 0.05).

Spleen is the secondary lymphoid organ that plays a crucial role in the host immune response against malaria parasite. During *Plasmodium* infection, infiltration of various lymphocytes occurs in the spleen, resulting in splenomegaly [30]. In addition, digestion of hemoglobin by the *Plasmodium* parasite during infection of red blood cells (RBCs) results in the formation of crystallized byproduct hemozoin (HZ) [31]. In this study, we determined the presence of hemozoin crystals in spleen sections of both *Plasmodium yoelii* 17XL and *Plasmodium yoelii* 17XNL infection (Figure 2A) at different time points post-infection. In spleen of uninfected control mice, a clear distinction between red and white pulp was seen along with resting follicles and marginal zones. However, in spleens of infected mice, an enlarged red pulp with increased cellularity and structure of white pulp was destroyed. Moreover, a distinct marginal zone surrounding the follicles became apparent with increase of infection. Compared to control, accumulation of hemozoin was observed in the in the pulp histiocytes and sinusoidal lining cells in spleen of both lethal and non-lethal parasite infected mice. However, in mice infected with lethal malaria parasite, *P. yoelii* 17XL, a constant increase in the accumulation of hemozoin crystals in spleen was noticed at various time points. On the contrary the concentration of hemozoin in spleen of *P. yoelii* 17XNL infected mice resolved with the decline of parasitemia (Figure 2A). It was interesting to note that the extent of hemozoin pigmentation in the spleen directly correlates with high parasitaemia. This histological variation in the red pulp, white pulp and vasculature of spleen of two different strains indicates the activation of a differential immune response.

Hemozoin was quantified in spleen of both group of mice infected with lethal Py17XL and non-lethal Py 17XNL malaria parasite spectrophotometrically at 400 nm (Figure 2B). The highest content of hemozoin was obtained in spleens of lethal parasite infected mice at 13 days post infection which appears to be correlated with increase of parasitaemia. Thus, with *Py* 17XL infection, we observed around 3.7 folds increase at 13th day compared to the 5th day of infection. However, in cases of non-lethal parasite infection, a completely different trend was observed. After achieving a peak in hemozoin content in mice infected with non-lethal parasite, the mean hemozoin content started declining until the 13th day post infection (Figure 2C). This decreasing trend in hemozoin content is indicative of the observed decrease in parsitaemia of mice infected with *Py* 17XNL.

### 3.3. Dynamics and Expansion of B Cells and IL-10 Producing Regulatory B Cells during Py 17XL and Py 17XNL Infection

**Figure 3:**
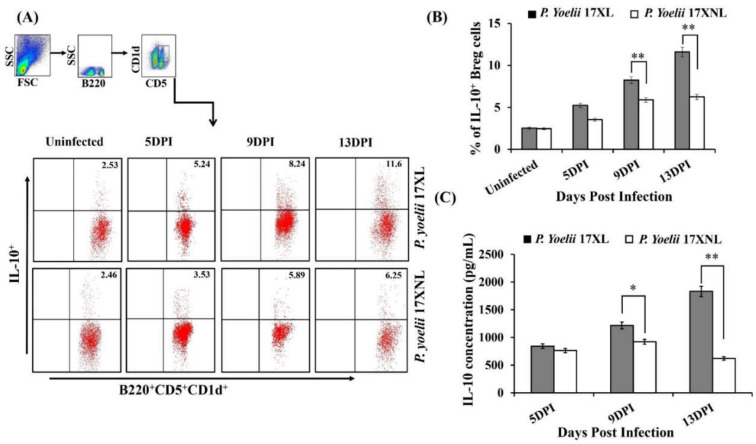
Immune dynamics of Bregs (regulatory B cells) during malaria infection with a lethal (*Py* 17XL) and non-lethal (*Py* 17XNL) strain of *Plasmodium*: Female Balb/c mice were intra- peritoneally (i.p) infected with 5 × 10^5^ *Plasmodium yoelii* 17XL and *Plasmodium yoelii* 17XNL parasitized Red Blood Cells. (**A**) Spleen cells from *Plasmodium* parasite infected and control mice were harvested and stained with fluorescently labelled antibodies against B220, CD5, CD1d and IL-10 at 5th, 9th and 13th days after infection of *Plasmodium yoelii* 17XL and *Plasmodium yoelii* 17XNL. Phenotypic characterization of regulatory B cells was done using flow cytometry. (**B**) The percentages of IL-10 producing B220^+^CD5^+^CD1d^+^ B cells in the spleen. (**C**) Serum profile of IL-10 cytokine after infection with *Plasmodium yoelii* 17XL *and Plasmodium yoelii* 17XNL at 5th, 9th and 13th day analyzed by using Luminex micro-bead array system. Data is representative of one of the three independent experiments. Unpaired two-tailed Student’s *t* test was used to calculate the statistical significance (** *p*  <  0.001, * *p* < 0.05).

We studied the dynamics of B220^+^ B cells during lethal *Plasmodium yoelii* 17XL and non-lethal *Plasmodium yoelii* 17XNL infection. The B220^+^ B cells showed a significantly increased number in the spleen tissue during *Plasmodium* infection when compared to uninfected mice. This rapid expansion indicates an important role of B cells during malaria pathogenesis in regulating the immune response. However, malaria-specific induction of regulatory B cells during lethal and non-lethal infection has not been studied to date and their role in the pathogenesis of malaria is still unclear. Therefore, we further studied the dynamics of regulatory B cells after *P. yoelii* 17XL and *P. yoelii* 17XNL infection at different time intervals including early, peak and late stages of parasite growth. The population of IL-10 producing B220^+^CD5^+^CD1d^+^ cells was found to be directly correlated with the increase of parasitemia (Figure 3A,B) in both lethal and non-lethal strains of *Plasmodium* at early stage of infection. Although, after reaching the peak on day 9 post-infection, the population of regulatory B cells did not increase further and started to decline rapidly in Balb/c mice infected with non-lethal *P. yoelii* 17 XNL strain. However, the frequency of these cells in Balb/c mice infected with *P. yoelii* 17XL kept on increasing until mice died.

To attain more understanding into the Breg cells mediated immune response against *Plasmodium* parasite, we determined the level of IL-10 cytokine in the serum of mice infected with *Py* 17XL and *Py* 17XNL. We found that *Plasmodium yoelii* 17XL infected mice produced significantly increased amounts of IL-10 as compared to *Plasmodium yoelii* 17XNL infected animals (Figure 3C). Further, to understand the physiological role of these cells, we performed an adoptive transfer experiment.

### 3.4. Adoptive Transferred Regulatory B Cells Mediating Protection

**Figure 4:**
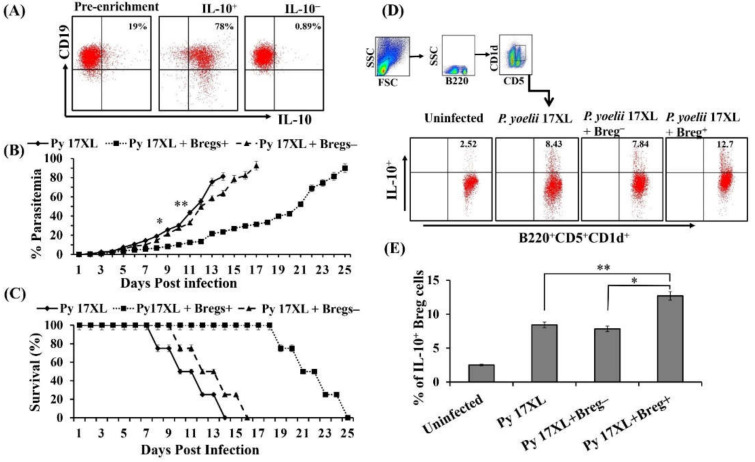
Adoptive transferred Bregs cells mediating protection: IL-10^+^ B cells were collected from *Plasmodium yoelii* 17XL infected mice. Isolated Breg cells were transferred via intra-venous *(i.v.)* into naive mice followed by *Plasmodium yoelii* 17XL infection. One group of animals received IL-10^+^ B (5 × 10^6^ cells) isolated from *P. yoelii* 17XL malaria-infected mice. The second group of mice was given IL-10^−^ B (5 × 10^6^ cells) isolated from *P. yoelii* 17XL malaria-infected mice. A third group of mice was infected with *P. yoelii* 17XL only without receiving any cells. These groups were compared with uninfected wild type control. (**A**) The purity of isolated IL-10^+^ and IL-10^−^ B cells was determined by using flow cytometry. (**B**) Parasitemia of mice that received IL-10^+^ B cells (square, *n* = 9) and IL-10^−^ B cells (triangle, *n* = 9) was determined daily over the course of parasite growth. Statistical significance was determined using student’s *t*-test (** *p* < 0.01, * *p* < 0.05) (**C**) Survival of mice receiving IL-10^+^ B cells or IL-10^−^ B cells in comparison to normal mice. Data is represented as mean ± SD from nine mice combined from three experiments performed independently consisting of three mice in each group. (**D**) The percentage of CD19^+^CD5^+^CD1d^+^ IL-10 producing regulatory B cells in spleen after 9th day of infusion of either IL-10^+^ B cells or IL-10^−^ B cells. Splenocytes were isolated at the 9th day after adoptive transfer and stained against B220, CD1d, CD5, and IL-10 with fluorescently tagged antibodies. (**E**) Bar graphs indicate the mean of percentages ± SD of splenic IL-10 producing B220^+^CD5^+^CD1d^+^ B cells. Single and double asterisks indicate *p* values of <0.05 and <0.01, respectively, determined by Student’s *t*-test.

We evaluated the immunomodulatory effect of regulatory B cells by performing adoptive transfer into naive mice which were subsequently infected with the malaria parasites. Considering the increased frequencies of Bregs in Balb/c mice infected with the lethal strain of *Plasmodium*, we isolated regulatory B cells from spleens of *Plasmodium yoelii* 17XL infected Balb/c mice on day 9 post-infection. Purity of isolated IL-10^+^ and IL-10^−^ B cells was determined using Flow cytometry after pre-enrichment of B cells (Figure 4A). These IL-10^+^ Breg cells were adoptively transferred (5 × 10^6^ cells) into naive mice followed by infection with the *Py* 17XL strain. As expected, adoptive transfer of regulatory B cells resulted in slowed parasite growth in recipient mice as determined by thin blood smears in comparison to mice receiving IL-10^−^ B cells, as shown in Figure 4B. We also observed a protective response leading to enhanced survival in mice infused with regulatory B cells as compared to only infected mice (Figure 4C). Further, to explore the immunological aspects of Breg induced regulation, we determined the frequency of regulatory B cells following adoptive transfer of Breg^+^ or Breg^−^ cells. For this, splenocytes were isolated by smashing spleen of *Py* 17XL infected mice infused with either Breg^+^ or Breg^−^ cells after 9th day of adoptive transfer followed by staining with B220, CD5 and CD1d along with IL-10 antibodies. Surprisingly, mice that received regulatory B cells exhibited profoundly increased numbers of IL-10 producing B220^+^CD5^+^CD1d^+^ cells than the control group of mice after 9th day of the adoptive transfer (Figure 4D,E). Data obtained from the study further confirmed the important immunoregulatory role of regulatory B cells against the *Plasmodium* parasite with increased survival and low parasitemia in Breg^+^ recipient mice.

### 3.5. Effect of Adoptive Transfer of Regulatory B Cells on Pro-Inflammatory Immune Response

**Figure 5:**
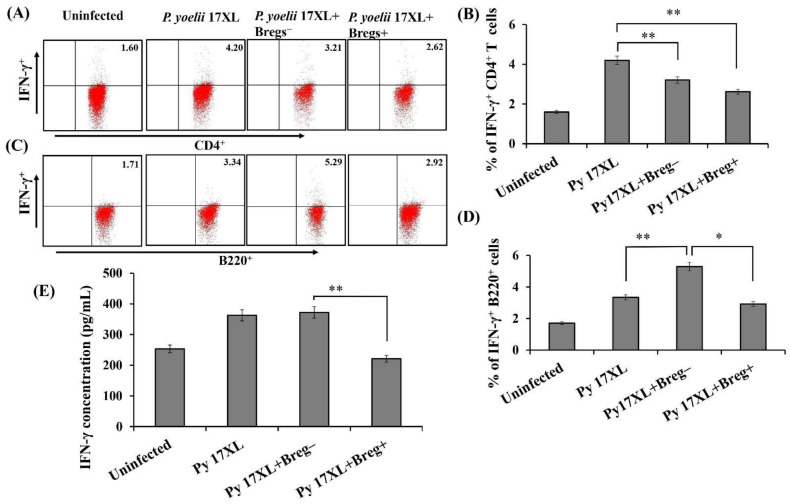
Adoptive transfer of regulatory B cells and its effect on pro-inflammatory immune response: Infusion of regulatory B cells modulates the immune response by altering IFN- γ cytokine profile. Splenocytes were harvested from spleen obtained at day 9 after infection from *Py* 17XL infected mice either intravenously infused with Breg^+^ or Breg^−^ cells. Harvested splenocytes were incubated with anti-IFN-γ, anti-B220 and anti-CD4 antibodies. (**A**,**C**) Flow cytometric analysis represents the IFN-γ expression on CD4^+^ T-cells and B220^+^ B cells in uninfected control, *P. yoelii* 17XL infected mice and *P. yoelii* 17XL infected mice either infused with either Breg^+^ cells or Breg^−^ cells (*n*  =  12). (**B**,**D**) Bar graph representing the percentage of IFN-γ on T cells and B cells. (**E**) Serum was harvested after 9 days of adoptive transfer of regulatory B cells in syngeneic Balb/c mice and IFN-γ expression was measured by Multiplex microarray bead-based system. Data is represented as mean ± SD of 9 mice combined from three independent experiments and unpaired two-tailed Student’s *t*-test was used to calculate statistical significance (* *p* < 0.05, ** *p* < 0.001).

Various pro-and anti-inflammatory cytokines are considered significant mediators of the immune response that determine the disease pathogenesis against malaria parasites. To elucidate the role of IL-10 producing B cells in the pathogenesis of malaria, IL-10^+^ B cells recipient mice after adoptive transfer were investigated for the expression of an important pro-inflammatory cytokine, IFN-γ, in spleen flow cytometrically. Splenocytes harvested from adoptively transferred mice were stained with surface markers CD4 and B220 along with IFN-γ antibodies. We noticed a significantly lower population of IFN-γ expressing CD4^+^ cells in IL-10^+^ Breg recipient mice in comparison to the control group of animals 9 days post infection (Figure 5A,B). To date, IFN-γ cytokine is mainly known to be produced by the T cell populations, recently a new sub-population of B-cells have been identified that express IFN-γ under some immunological conditions during certain infections, although not very well characterized in malaria [32,33,34]. However, we observed almost similar percentage of IFN-γ expressing B220^+^ cells in spleen of Breg^+^ recipient mice when compared to Breg^−^ recipient mice or only *Py* 17XL infected mice (Figure 5C,D). Further, serum cytokine profile also showed a significant decrease in level of this pro-inflammatory cytokine IFN-γ (Figure 5E). Overall, our data indicates the immunosuppressive effect of regulatory B cells during *Plasmodium* infection by counteracting the excess of pro-inflammatory cytokine that ultimately results in inhibition of surplus pro-inflammatory response.

### 3.6. Effect of Adoptive Transfer of Regulatory B Cells on Anti-Inflammatory Immune Response

**Figure 6:**
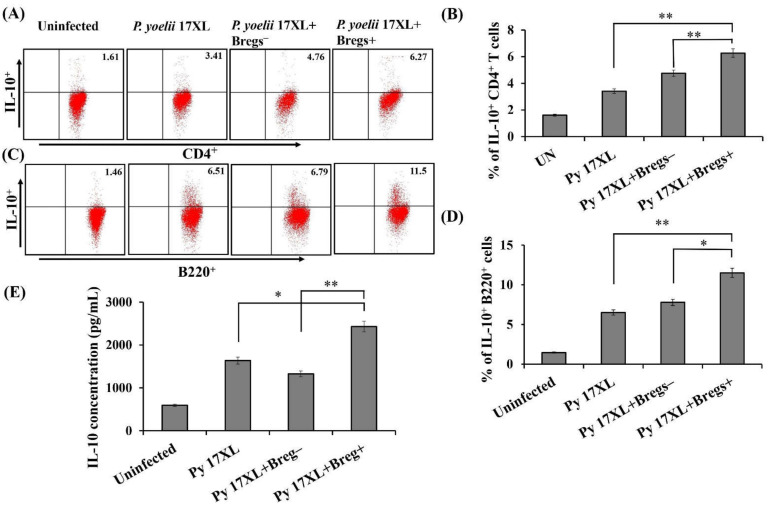
Adoptive transfer of regulatory B cells and its effect on anti-inflammatory immune response: Infusion of regulatory B cells modulates the immune response by altering cytokine profiles. Splenocytes were harvested at day 9 post-infection from *Py* 17XL infected mice either infused with Breg^+^ or Breg^−^ cells. Splenocytes were stained with IL-10 specific antibody along with anti-B220 and anti-CD4 antibodies. (**A**,**C**) Flow cytometric analysis shows the expression of IL-10 cytokine on CD4^+^ T-cells and B220^+^ B cells in uninfected, *P. yoelii* 17XL infected mice and *P. yoelii* 17XL infected mice either infused with Breg^+^ cells or Breg^−^ cells (*n*  =  12). (**B**,**D**) Bar graph representing the percentage of IL-10 on T cells and B cells. (**E**) Serum samples were collected after 9th day of adoptive transfer of regulatory B cells. IL-10 expression was measured by Luminex micro-bead array system in *Py* 17 XL infected mice infused with Breg^+^ cells or the Breg^−^ cells. Data are shown as mean ± SD of 9 mice pooled from three independent experiments statistical significance was determined by unpaired two-tailed Student’s *t*-test (* *p* < 0.05, ** *p* < 0.001).

Various studies have associated the role of cytokine IL-10 in regulating the detrimental effects of most pro-inflammatory cytokines. To unravel the role of IL-10 producing B cells in the pathogenesis of malaria, IL-10^+^ regulatory B cells recipient mice were investigated for anti-inflammatory immune response. After adoptive transfer, dynamics of CD4^+^IL-10^+^ cells were significantly increased in Breg^+^ recipient mice (Figure 6A,B). We also noticed that IL-10^+^ Breg recipient mice had a relatively higher population of IL-10 producing B cells than the control group of mice after 9 days of the adoptive transfer (Figure 6C,D). Further, analysis of serum from Breg recipient mice showed a higher concentration of IL-10 cytokine compared to non-Breg recipient group (Figure 6E). Taken together, our results indicate that the immunosuppressive effect of regulatory B cells during *Plasmodium* infection is mediated by enhancing the production of anti-inflammatory response.

### 3.7. Effect of Adoptive Transfer of Bregs on PD-L1 Expression

**Figure 7:**
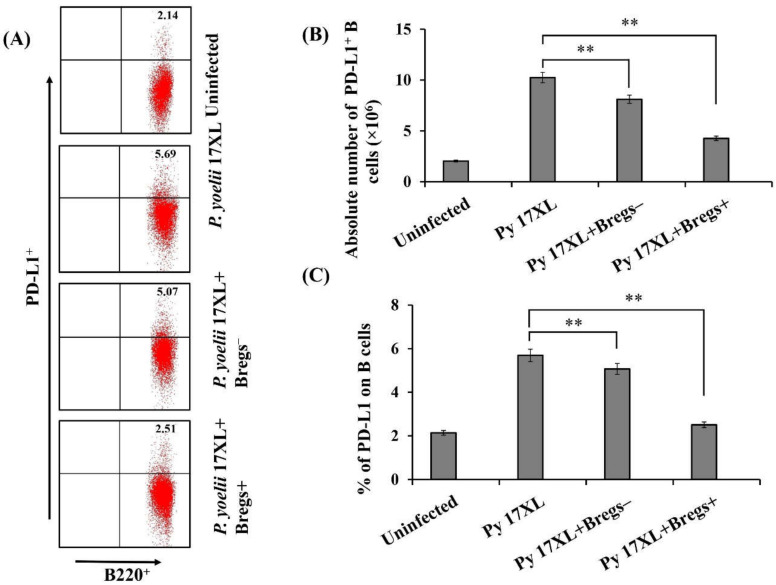
Effect of adoptive transfer of regulatory B cells on the expression of PD-L1: Splenocytes were harvested from *Py* 17XL infected mice infused with IL-10^+^ B cells or IL-10^−^ B cells, only infected and normal uninfected control. (**A**) Spleen cells were stained against PD-L1 andB220 using fluorescent tagged antibodies. Splenocytes were gated on whole lymphocytes to determine PD-L1^+^ B cells. (**B**) Absolute of PD-L1^+^ B cells after adoptive transfer of Bregs (**C**) Percentage of PD-L1 expressing B220^+^ B cells in spleen of *Py* 17XL infected mice infused with either Breg^+^ or Breg^−^ cells, only *Py* 17XL infected and uninfected mice.(** *p* < 0.001).

Programmed death ligand-1 (PD-L1) is an important member of the B7 family. PD-L1^hi^ has the ability to control immune function by binding to PD-1 thereby inhibiting activation of T cells. Therefore, after the adoptive transfer of regulatory B cells, we examined the expression of PD-L1 on B cells. It was observed that regulatory B cells recipient mice have significantly low expression of PD-L1 compared to *Plasmodium*-infected mice. Therefore, adoptive transfer of regulatory B cells resulted in decreased expression of PD-L1 indicating resistance to the *Plasmodium* parasite (Figure 7A,C). Absolute numbers of PD-L1-expressing B220^+^ B cells also showed a significant decrease after adoptive transfer compared to control group of animals (Figure 7B).

## 4. Discussion

The cytopathology of severe and non-severe forms of malaria is largely determined by the immunomodulation induced as a result of parasite–host interaction [35,36]. The outcome of induced immune response is the cumulative effect of both innate and adaptive immunity [37]. Nevertheless, one of the critical roles in determining the severity of infection is played by the species of *Plasmodium* parasite involved as well as the stage of infection. In general, different immune cells are involved in generating an effective immune response and typically the pathogenesis of malaria is characterized by pro-inflammatory cytokines at an initial stage of *Plasmodium* infection [11,38,39]. Besides T cell response, B cells are considered a very critical part of the host-mediated immune response by differentiating themselves into antibody-secreting plasma cells [40]. It has been reported that mice deficient in B lymphocytes are not able to eliminate the blood stage of the parasite and generate protection [41]. Recently, an important novel sub-population of B cells, called regulatory B cells, was identified and are supposed to be involved in immune regulation mediated by immunosuppressive cytokines [23,42]. To date, regulatory B cells have been described in several studies involving human and murine models under various disease conditions [22,43]. Although studies demonstrate the emergence of regulatory B-cells as an important immunoregulator, its role in lethal and non-lethal malaria infection is limited. In this study, we performed a comparative analysis of regulatory B cells mediated immune response during infection by lethal *P. yoelii* 17XL and non-lethal *P. yoelii* 17XNL murine malaria parasites. Regulatory B cells are known to moderate immune associated pathology by suppressing inflammatory immune response [44]. Generally, these cells are involved either in development of disease or for generating resistance against the disease depending on the immune response generated against the pathogen. Despite the absence of studies directly investigating the role of regulatory B cells, several groups have reported that the rapid expansion of B cells occurs during infection with *Plasmodium* parasite in humans [45,46]. The results from the current study showed a rapid expansion of B220^+^CD5^+^CD1d^+^ IL-10 producing regulatory B cells during infection with both lethal strain *P. yoelii* 17XL and non-lethal *P. yoelii* 17XNL at an early stage of infection. Although, infection with the lethal strain showed a rapid and continuous increase in expansion of these cells with the growth of parasite. Data indicates that regulatory B cells are actively induced during infection and might be able to maintain host-parasite equilibrium during infection. Cytokine analysis also showed an increased expression of IL-10 during *Py* 17XL infection indicating an immunosuppressive mechanism against excessive proinflammation. The delicate balance between pro-and anti-inflammatory cytokines immune response has a huge effect in determining the outcome of *Plasmodium* infection. There is substantial evidence to demonstrate the immunomodulatory role of these regulatory B cells in susceptibility or resistance under several disease conditions. In our study, adoptive transfer of regulatory B cells following infection with a lethal parasite resulted in enhanced survival of *Py* 17XL infected mice and slowed the growth of the *Plasmodium* parasite. We also observed a considerable increase in the number of IL-10 producing B220^+^CD5^+^CD1d^+^ cells in the recipient mice after adoptive transfer. This implies that these regulatory B cells may be providing immune protection by preventing the inflammation associated with disease, thereby inhibiting the parasite growth. Although, only minor difference was observed in the enhancement of survival of *Plasmodium yoelii* 17XL infected mice after adoptive transfer. Thus, it can be assumed that the immunosuppression induced by Bregs might be utilized by the host to build a favorable microenvironment resulting in inhibited growth of parasite.

Further, we studied the expression of pro- and anti-inflammatory mediators in the spleen of adoptively transferred mice. Our results showed a significant decrease in pro-inflammatory cytokine IFN-γ in adoptively transferred mice compared to control group of animals, indicating the immunosuppressive effect of regulatory B cells. The immunosuppressive effects of Bregs were further validated by analyzing the expression of IL-10 cytokines by immune cells. The exuberated production of major anti-inflammatory cytokine (IL-10) by immune cells such as B and T cells confirms our hypothesis of immunosuppression-mediated protection of Bregs. The results obtained are in accordance with the fact that to confer protection against the pathogen, it is important to counterbalance the excessive pro-inflammatory response by enhancing the immunosuppressive anti-inflammatory response. Therefore, the outcome obtained is very consistent with the earlier reported explanations, namely, that an intricate balance in the levels of both pro-and anti-inflammatory mediators determines the outcome of disease towards susceptibility or resistance.

## 5. Conclusions

In conclusion, we have shown that regulatory B cells are actively induced during *Plasmodium yoelii* 17XL infection in the spleen. Adoptive transfer of IL-10^+^ Breg cells resulted in improved survival of the infected mice and inhibited growth of *Plasmodium* parasite. Further evidence implies that Breg cells may serve as an important contributor in limiting the severe pro-inflammatory immune response owing to their pleiotropic effects in stimulating IL-10 expression. Taken together, it can be concluded that regulatory B cells, being an important mediator of immunoregulation, are a vital consideration in determining the outcome of malaria infection. These novel findings will serve as key point in understanding the overall physiology of malaria pathogenesis.

## Data Availability

Not applicable.
